# Use, Practices and Attitudes of Elite and Sub-Elite Athletes towards Tart Cherry Supplementation

**DOI:** 10.3390/sports9040049

**Published:** 2021-03-31

**Authors:** Jimmy T. Wangdi, Vlad Sabou, Mary F. O’Leary, Vincent G. Kelly, Joanna L. Bowtell

**Affiliations:** 1 School of Human Movement and Nutrition Sciences, University of Queensland, Brisbane, QLD 4072, Australia; j.wangdi@uq.net.au (J.T.W.); v6.kelly@qut.edu.au (V.G.K.); 2 Sport and Health Sciences, St Luke’s Campus, University of Exeter, Heavitree Road, Exeter EX1 2LU, UK; vs348@exeter.ac.uk (V.S.); M.OLeary@exeter.ac.uk (M.F.O.); 3 School of Exercise and Nutrition Sciences, Queensland University of Technology, Brisbane, QLD 4000, Australia

**Keywords:** exercise performance, exercise recovery, sleep, polyphenols, ergogenic aids, athletes

## Abstract

Tart cherry (TC) supplementation can improve exercise recovery and performance; and may also improve sleep duration and quality. This study investigated the use and knowledge of TC supplementation by athletes of all competitive levels. Eighty participants (52.5% elite (international, national, professional), 47.5% sub-elite (semi-professional, state/regional, county level, club level, recreational)) completed an online questionnaire investigating their attitudes towards and use of TC supplementation. Overall, 22.6% of participants were using or had previously used TC supplements, and 12.5% of participants planned to used TC supplements. Improved recovery (71.4%), sleep (32.1%) and immunity and general health (32.1%) were the most frequently indicated goals by respondents using TC supplements. In total, 32.1% of respondents were supplemented with TC chronically, 39.3% acutely and 28.6% used a combination of chronic and acute supplementation. The majority of those employing TC supplementation chronically used TC either over 2–3 days (37.0%) or continuously (37.0%). The most popular TC pre- and post-loading period was one day (34.3% and 41.5%, respectively). There were no significant differences between elite and sub-elite athletes in any parameters assessed (*p* > 0.05). TC supplementation is not widely used by the athletes surveyed, and athletes using TC supplements showed poor awareness of an evidence-led dosing strategy, regardless of competitive level.

## 1. Introduction

Recent publications have shown supplementation of tart cherry (TC) in multiple forms can improve recovery from exercise induced muscle damage [1,2,3,4,5,6,7]; exercise performance [8,9]; and sleep quality [10,11,12]. These ergogenic effects have been linked to TC’s high concentrations of phenolic acids and flavonoids such as anthocyanidins [13], which are involved in a number of important processes within the plant, most notably ultraviolet screening to protect against ionising solar radiation and subsequent reactive oxygen species (ROS) production [14,15,16].

The protective antioxidant properties of these compounds are likely to have been the primary initial driver of research into their use as a supplement to reduce oxidative stress and inflammation. Whilst the mechanisms of action are currently unclear, supplementation with polyphenols has been shown to reduce oxidative stress; this has multiple implications for health including reduced risk of diabetes, cardiovascular disease and osteoarthritis [17,18,19,20], as well as improved exercise recovery and performance [1,2,3,4,5,6,7,21]. Furthermore, they may also have anti-inflammatory properties. Polyphenols can inhibit pro-inflammatory cyclooxygenase enzyme activity (COX1 and COX2) [22,23,24,25]. Their ability to inhibit COX2 is most notable, as this is a key mechanism of endogenous antioxidant defence [26]. Inhibition of pro-inflammatory cytokines can help to reduce inflammation in skeletal muscle following exercise [27], which is also likely to aid recovery from muscle damage. As well as antioxidants, TC contains high levels of melatonin [28], which is associated with sleep promotion [29,30]. Indeed, TC has been shown to increase the total sleep time, as well as quality, though this has only been shown in healthy and active adults in one study [10].

Despite this growing body of evidence, and increasing number of commercially available TC supplements, little is known of the application of these findings in applied settings as it relates to the supplementation behaviour, beliefs and perceptions of athletes. Knowledge around supplement use, including factors such as the appropriate dose, period of supplementation and objective of supplement use, are important factors for an effective supplementation strategy. In both elite and sub-elite sports, nutritional interventions are often employed [31], perhaps due to the relative ease of dietary manipulation compared to other interventions. However, athlete knowledge of and understanding around the use of supplements has previously been shown to be poor [32,33,34]. Furthermore, the use and practices of TC supplementation by sports science practitioners has also been shown to be divergent from the literature [35], and thus we hypothesized that similar findings may be uncovered in athletes, many of whom, in elite settings, are likely to be provided with information and supplementation strategies from sports science practitioners.

The aim of this study was to investigate the use of TC supplementation by athletes of all competitive levels, to assess their pre-existing knowledge of TC and awareness of potential benefits of use, and to assess the degree to which supplementation strategies are evidence led.

## 2. Materials and Methods

Eighty participants (51 males, 27 females, 2 rather not say, age = 27.6 ± 9.8 years, weight = 74.2 ± 15.0 kg, height = 176.8 ± 12.1 cm), competing at a range of levels (international and professional to recreational), from 15 countries (Afghanistan, Australia, Brunei Darussalam, Canada, the Czech Republic, Ireland, Italy, Latvia, Lithuania, Nepal, Oman, South Africa, Switzerland, the United Kingdom of Great Britain and Northern Ireland, and the United States of America) across 21 sports (archery, athletics/track and field, basketball, Brazilian jujitsu, CrossFit, cycling, duathlon, football (soccer), hockey, long track speed skating, netball, paracanoe sprint, powerlifting, rowing, rugby union, running, strength training, tennis, triathlon, weaselling, weight-lifting and no primary sport/just recreational exercise) were recruited through social media and posters, and completed at least 2 sections of a survey on their use of TC supplements. Individuals who completed less than 4 h of exercise per week were excluded. Participants gave informed consent and completed an online questionnaire of up to 48 questions. The study was approved by The University of Exeter Sport and Health Sciences Research Ethics Committee.

The questionnaire was conducted anonymously using an online survey platform (Qualtrics) that conformed with General Data Protection Regulation (GDPR) laws, and included questions based on or adapted from questionnaires used in previous research investigating the usage and knowledge of a dietary supplementation in athletes [34,36,37]. The aims of these questions were to gather information on participant demographics, their knowledge and opinions of TC, their current or previous use of TC, and their sources of information on TC. Questions were predominantly presented as multiple choice; however, some required a written response, and for some questions multiple answers could be provided. An “other” option allowing additional answers to be provided was available for the majority of the multiple-choice questions. For many of the multiple-choice questions assessing knowledge and awareness, incorrect options were also included to ensure options were selected based on prior knowledge. The questionnaire was set up to skip non-relevant questions based on previous answers.

Questions were separated into sections, with 13 questions assessing participant demographics; 23 questions assessing participant use of TC; and 12 questions assessing participant attitudes towards TC supplements. A full copy of the questionnaire is available in the supplementary material S1. Questions in Section S1 consisted of gathering information on participant characteristics, as well as information on competitive level, sport or sports competed in, the type of training they undertake and the amount of time they spend training and competing.

In Section S2, participants were asked questions about their knowledge, and use of TC supplements. Questions centred around sources of information, beliefs about TC supplements, goals of supplementation and supplementation practices. The questions were comprised of a combination of 18 multiple choice questions, 11 of which also provided participants with the option to write in another answer, and 4 questions where participants were asked to write in answers.

In Section S3, participants were asked what performance outcomes/benefits they believe the current TC literature supports, how this has informed their supplementation, and if they had noticed any effects personally. This section contained 4 multiple choice questions, 2 of which had the option to write in another response, and three questions where participants were asked to write in answers.

Data were analysed to provide descriptive statistics and frequencies for demographic data. Chi squared analysis was also performed to detect differences between answers of athletes at different competitive levels. For questions assessing the evidential support for their beliefs in the effects of TC supplementation, answers were classified as ‘evidence led’, or ‘non-evidence led,’ and participants were categorised into either ‘only evidence led answers selected’, or ‘non-evidence led answers selected.’ Participants’ sources of information were categorised into professional and non-professional, to allow assessment of professionality of the source of their information. All data were analysed using SPSS (version 24, IBM, Armonk, New York, NY, USA).

## 3. Results

### 3.1. Participant Demographics

Eighty respondents met the eligibility criteria and completed at least two of three questionnaire sections. The mean number of hours participants spent training per week was 13.5 ± 7.9 h. Information on participant training and competition demographics can be found in Table 1.

### 3.2. Use of Tart Cherry Supplements

**Figure 1:**
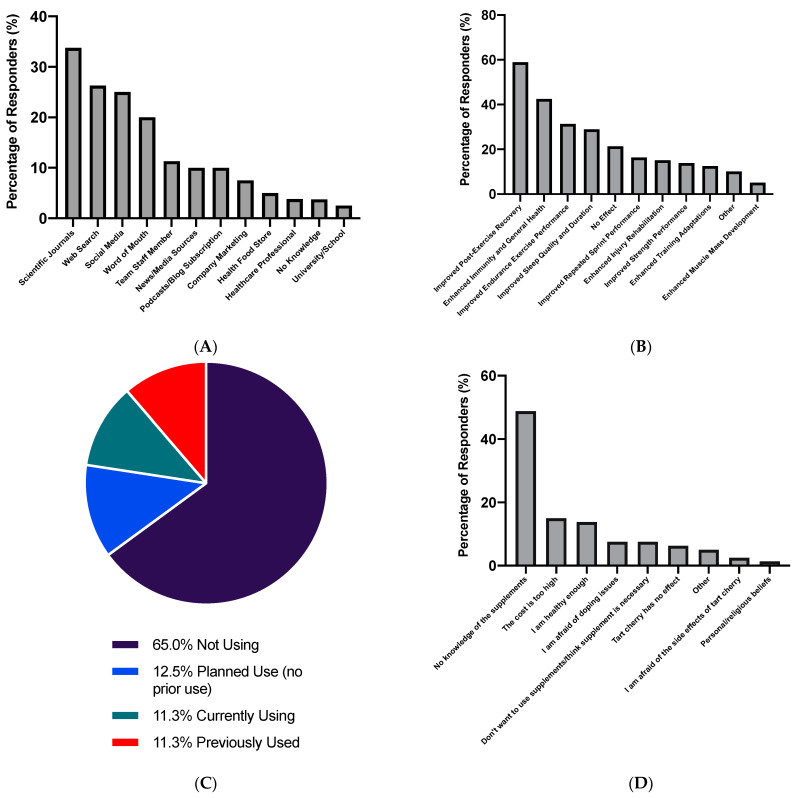
Participant responses for (**A**) sources of information on cherry supplements (**B**) awareness of potential benefits of cherry supplementation (**C**) use of cherry supplements (**D**) reasons for not using cherry supplements. Values are displayed as percentage of responses (*n* = 80).

When all respondents (*n* = 80) were asked to rate their pre-existing knowledge of TC supplements, 7.5% of respondents indicated their knowledge was excellent, 21.3% good, 12.5% average, 18.8% limited and 40.0% indicated they had no knowledge. Participant sources of information are displayed in Figure 1A, and participant awareness of potential benefits is displayed in Figure 1B. Of the 10.0% of participants who answered ‘other,’ 5.0% indicated they were unsure of the potential benefits, 1.3% (*n* = 1) decreased soreness, 1.3% a reduction in uric acid during adaptation to a ketogenic diet, and 1.3% provided no elaboration. Participant use of TC supplements is displayed in Figure 1C, and reasons for not using TC supplementation are displayed in Figure 1D. Of the 5.0% of participants who selected ‘other’ as an option, 2.6% indicated they were not competing at a high enough level to think use necessary, 1.3% indicated it was due to other supplement use, and 1.3% due to a lack of evidence for TC supplementation’s effectiveness.

Those individuals who earlier indicated they previously used or were currently using TC supplements (22.6%, *n* = 18), were then asked when they last used a TC supplement; 27.8% of indicated they had in the past 7 days, 16.7% in the past 30 days, 27.8% in the past 1–6 months, 16.7% in the past 7–12 months, and 11.1% more than 1 year ago. When asked at what competitive level these respondents (*n* = 18) first used TC supplements 16.7% indicated international level, 27.8% national level, 5.6% (*n* = 1) professional level, 11.1% semi-professional level, 5.6% state/regional level, 11.1% county level, 11.1% club level, and 11.1% recreational level.

**Figure 2:**
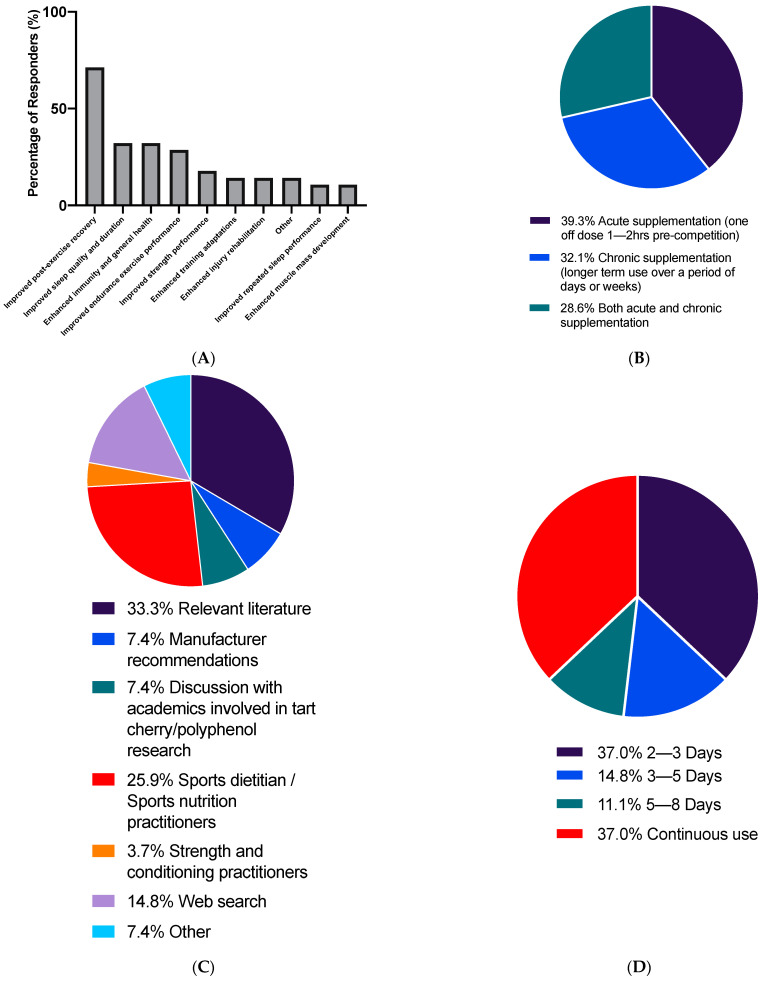
Participant responses for (**A**) Goals of supplementation of TC supplements (*n* = 28) (**B**) how they do, did or would use TC supplements are displayed in figure (*n* = 28) (**C**) what was the main source of they would use to inform their TC supplementation protocol (*n* = 27). (**D**) what is, was or would be the usual duration of their TC supplementation (*n* = 27).

The goals of TC supplementation for respondents who had previously used, currently use or plan to use TC supplements (*n* = 28) are shown in Figure 2A. Of the four participants who answered ‘other’, responses were to fix joint pain from high uric acid, reduce illness and improve recovery speed from sessions, to prevent inflammation, and one participant indicated they were not using the supplement yet. When all respondents (*n* = 80) were asked what type of TC supplement they use or would use, 40.0% indicated TC concentrate juice, 38.8% TC juice, 27.5% TC powder, 28.8% TC gel, 8.8% other. Of the 8.8% (*n* = 7) of participants who answered other, two participants indicated some form of tablet, and the remaining five indicated they were unsure or could not answer because they do not plan on using the supplement. Of participants who had used, currently use or plan to use the supplement (*n* = 28), 57.1% indicated they use TC concentrate juice, 50.0% TC juice, 25% TC powder, 14.3% TC gel, 3.6% tablets.

The TC supplementation protocols for participants, who had used, currently use or plan to use TC supplements (*n* = 28 are displayed in Figure 2B. Responses regarding duration of their supplementation are described in Figure 2D (*n* = 27), and the main source of their TC supplementation protocol information in Figure 2C (*n* = 27). Of the 7.4% (*n* = 2) of participants that answered other in the last of these questions, one indicated the cost of the supplement played a role in their decisions about supplementation protocol, and one consulted with others.

**Figure 3:**
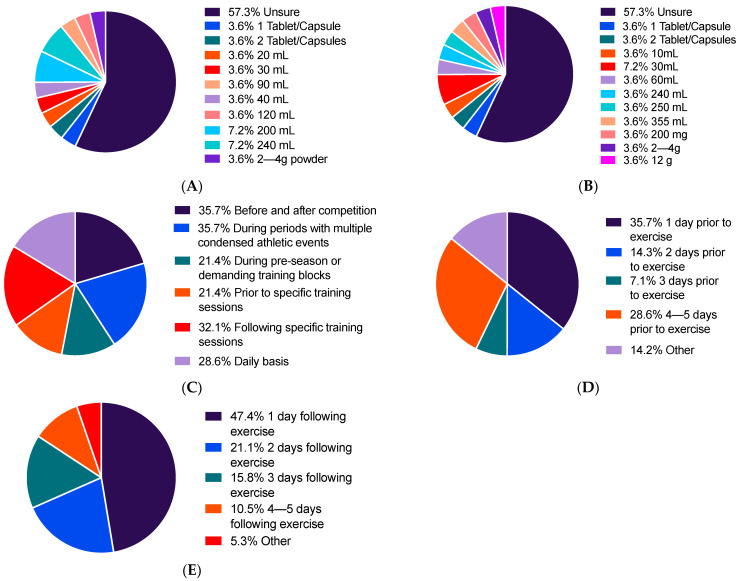
Participant responses for (**A**) dose used during chronic TC supplement use (*n* = 27); (**B**) dose used during acute TC supplement use (*n* = 27); (**C**) periods during which TC supplementation are/were/would be primarily used (*n* = 28); (**D**) the pre-load supplementation period employed when using TC supplementation for recovery (*n* = 14); (**E**) the post-load supplementation period employed when using supplementation for recovery (*n* = 19).

When participants who had used, currently use or plan to use the supplement (*n* = 27) were asked what dose they use or would use for both chronic and acute supplementation periods; the majority of participants were unsure (Figure 3A,B). When asked if they were aware of the polyphenol content within this dose, only one participant for chronic supplementation and two participants for acute supplementation were aware of the polyphenol dose provided by supplementation, with the majority again selecting ‘unsure’ or not answering the question. These same participants’ answers as to what periods they would use TC supplementation for are displayed in Figure 3C. Participants were then questioned on the duration of their pre-load (Figure 3D) and post-load (Figure 3E) supplementation protocol, if they had previously indicated they used, use or would use TC supplementation for recovery. Of the participants who answered ‘other’ for pre-load, 7.1% (*n* = 1) indicated they took the supplement ~1 h post competition and 7.1% indicated they take the supplement daily. The participant (5.3%, *n* = 1) who answered ‘other’ for post-load indicated they take the supplement daily.

For participants who had used or currently use TC supplements (*n* = 18) answers to what side effects they experienced, and the severity of the side effects following supplementation are displayed in Table 2 (*n* = 18). All participants that indicated other for this question answered ‘N/A’ or that they had experienced no side effects.

### 3.3. Attitudes towards Tart Cherry Supplements

Of all respondents (*n* = 80), 26.0% indicated there was support within the literature for improved post-exercise recovery, 15.0% for improved sleep quality and duration, 11.3% for improved exercise performance following acute supplementation, 8.8% for enhanced immunity, 7.5% for enhanced injury rehabilitation, and 40.0% indicated they were unsure. Meanwhile, 33.8% indicated they believe the literature supports efficacy of supplementation for endurance-based sports, 15.0% for strength-based sports, 15.0% for intermittent-running based sports/team sports, 12.5% for power-based sports, 7.5% none of the above, 27.5% indicated they were unsure and 3.8% other. Of those respondents who answered ‘other’ 1.3% (*n* = 1) indicated any sport, 1.3% indicated they did not know, and 1.3% that they believed benefits would be seen for any sports in which reducing delayed onset muscle soreness (DOMS) would be advantageous. When respondents who had previously used or currently use TC supplements (*n* = 15) were asked if they had experienced improvements in performance following TC supplementation 80.0% indicated they had not, with 20.0% indicating they had; 60.0% indicated they had not experienced any improvement in recovery, whilst 40.0% indicated they had. Of these participants (*n* = 14), 85.7% also indicated they had not experienced any improvements in sleep, with 14.3% indicating they had experienced sleep improvement. When all participants (*n* = 57) were asked how they believed TC supplementation may influence improvements from training, 42.1% indicated they thought it would be likely to have a positive effect, 3.5% thought it would be likely to have a negative effect, 13.8% it would be likely to have no effect, and 35.1% indicated they had no opinion on the question. When the same respondents were asked if they would use TC supplementation and how, if it was shown to impair improvements from training, 78.1% indicated they would not use it, 5.3% that they would still use it consistently, 3.5% that they would use it for one off acute doses to improve performance, 1.8% would use chronic doses during specific periods of training/off season, 5.3% would use chronic doses during specific periods of competition and 7.0% indicated ‘other.’ Of those participants that indicated ‘other’ 1.8% (*n* = 1) indicated they were aware of the potential impairments to training, and thus currently use the supplement during periods where adaptation is less important than recovery, and 1.8% indicated that they use if for sleep and thus would continue to do so.

When participants who had previously used, currently use, or plan to use TC supplements (*n* = 28) were asked what their biggest considerations were when deciding how to develop their TC supplementation protocol 28.6% answered they considered the mixed findings regarding efficacy in the current literature, 25.0% indicated they considered the lack of clarity for an optimal supplementation strategy with regard to duration and dose, 17.9% indicated they considered the lack of research illustrating the effects on training adaptations, 3.6% (*n* = 1) indicated they had difficulties in choosing between available market products, 14.3% indicated they considered the supplement pricing. No participants indicated they considered prioritisation of other supplements, and 3.6% indicated ‘other’ considerations (*n* = 28) as factors in their decision. The participant who answered ‘other’ indicated they used prior knowledge of anthocyanins to develop their protocol.

Of participants who had used or currently use TC supplements, and who completed this question (*n* = 16), 68.8% responded that they enjoy the taste, 18.8% that they do not enjoy the taste, and 12.5% indicated they had no strong feelings on the taste. Overall, 43.8% of these participants indicated that the taste influenced their decision to use the supplement, and 56.3% indicated it did not influence their decision to use the supplement. Total participants who completed this part of the questionnaire (*n* = 54) indicated they would like to see; a beverage (25.9%), a concentrate (35.2%), a juice (22.2%), and a bar (7.4%) developed in the future and a further 9.3% indicated they would like to see ‘other’ supplements developed. Of those who answered other 1.9% (*n* = 1) indicated they did not mind, 1.9% indicated something easy to ingest, 1.9% indicated powder, 1.9% indicated tablets and 1.9% indicated they saw no need for further product development. When all respondents (*n* = 49), were asked to elaborate on their reason for indicating a particular supplement, 63.3% indicated the ease of consumption or convenience of use, 14.3% indicated the taste, 14.3% indicated that it was their preferred method of supplement consumption generally, 2.0% (*n* = 1) indicated they believed that form of supplement (bar) would reduce GI distress, and 2.0% indicated that they believe this form would reduce the CHO intake from supplementation (concentrate). Meanwhile, 4.1% of respondents indicated they would not use the supplement in any form.

### 3.4. Sub-Group Analysis: Elite vs. Sub-Elite

Sub-group analysis was performed using chi-squared tests to compare elite to sub-elite respondents. Respondents (*n* = 80) were classified as elite if their highest competitive level was international (20.0%), national (30.0%) or professional (2.5%); and sub-elite if their highest competitive level was semi-professional (2.5%), state or regional (12.5%), county level (2.5%), club level (16.3%) or recreational (12.5%). The first use of TC supplements by athletes who had used or were using TC supplements was 50% at elite and 50% at sub-elite level. The proportions of elite and sub-elite athletes who had used or use TC supplements (*n* = 18) was 61.1% indicating their highest competitive level as elite, and 38.9% sub-elite, with 11.1% of the elite athletes having first used TC supplements at a sub-elite level.

There was no significant difference between elite and sub-elite participants in self-assessed knowledge (*p* = 0.568, *n* = 80); in dose used for both chronic (*p* = 0.376) or acute (*p* = 0.279) supplementation protocols (*n* = 27); or in pre- (*p* = 0.856, *n* = 14) or post-load (*p* = 0.277, *n* = 19) supplementation period. There was no significant difference between elite and sub-elite participants in the evidential support for their beliefs in the effects of TC supplementation (*p* = 0.419, *n* = 79), or in the professionality of the source of their information (*p* = 0.555, *n* = 46).

## 4. Discussion

This study investigated the current use, practices and attitudes of athletes from a variety of competitive levels on tart TC supplementation. The study included individuals from 21 different primary sports competing in 15 countries. Overall, 22.6% of respondents were using or had previously used TC supplements, and 12.5% had plans to use them in the future. Athlete knowledge of the supplement was a major issue identified, with almost half of athletes reporting lack of knowledge of the supplement as the reason for not using TC supplementation.

Indeed, we also found a lack of knowledge of the supplementation protocol required, which is perhaps unsurprising given that this is an area within the literature where there is a dearth of knowledge. From those individuals who indicated a preferred TC dose, there was a large variation in doses. Duration of TC use was broadly distributed evenly between chronic use, acute use and a combination of both chronic and acute use. The majority of those employing TC supplementation chronically used TC either over 2–3 days (37.0%) or continuously (37.0%), which is surprising, as the majority of studies in the TC literature use supplementation periods of between 6 and 10 days [2,3,4,6,38,39,40]. Indeed, no research has been conducted on continuous TC use over an extended period of time, and we are aware of only one study that used a three day supplementation protocol [41] which showed no significant effects of supplementation on amelioration of reduced muscle function or muscle soreness. This suggests that many athletes may not be using an appropriate supplementation protocol to elicit ergogenic effects. Furthermore, whilst there is no evidence regarding the effects of polyphenol supplementation on adaptive responses to training, there is evidence to suggest that chronic supplementation of antioxidants may blunt such responses [42,43,44,45]. However, the predominance of this research has investigated vitamin antioxidants, which research suggests work via direct scavenging of free radicals [46,47]. Currently, there is no research showing decrements to the adaptation following supplementation of whole fruit polyphenol-based supplements, which are thought to exert antioxidant effects via a hormetic response [48]. There was also a great deal of variation between pre and post load durations, with the most popular duration for both being only one day (35.7% and 47.4%, respectively). Similarly, to overall supplementation duration, this does not align well with the literature, where pre-load periods range from 4–7 days and post loads range from 2 to 4 days [2,3,4,6,38,39,40].

Furthermore, when asked if they knew the polyphenol content of their supplement dose, only one participant using TC chronically and two participants using TC acutely were aware of the polyphenol dose they were taking. We have shown that many athletes lack knowledge of both the polyphenol content provided by their supplementation strategy and the polyphenol dose required to elicit benefits. Therefore, participants are likely to either under-dose and thus not elicit any beneficial effects, or over-dose, which may be undesirable from a cost–benefit perspective. This may explain why 80% of participants reported no performance benefits and 60% reported no improvement in recovery.

The lack of knowledge surrounding TC supplementation protocol and dose among our cohort is unsurprising due to the unclear nature of both dose and supplementation protocol within the literature. Across studies investigating the effects of supplementation on recovery and performance there are a multitude of different supplementation protocols encompassing both different pre-load and post-load lengths, different doses, and different supplement timings. Furthermore, many studies investigating recovery do not report dose timings at all [1,4,38], and thus it is impossible to quantify what differences are due to the acute versus the chronic supplementation period. The multitude of different supplementation protocols used in applied settings is also something that was shown when sports science practitioners were surveyed to assess their use and practices of TC supplements [35].

It is not clear how supplement modality may affect the efficacy of supplementation; this requires further research. Indeed, the majority of studies that show positive effects of supplementation use a juice or juice concentrate [1,2,3,4,5,6], with powdered fruit concentrate supplements appearing to be less effective [40,49]. This perhaps explains the majority of participants who use or have used TC supplements using a concentrate (57.1%); however, 25% of participants of this participant cohort also indicated that they used powdered supplements, and 27.5% of all respondents indicated TC powder would be their preferred form of supplement. This suggests that athletes may be unaware of potential discrepancies in efficacy between concentrates and powders. This difference between supplementation types may perhaps be due to differences in production methodology. It is likely that the processing of the cherries differs between production of powder and juice, which may increase variation in the polyphenol blend, above that which would occur naturally between batches of a given product. Indeed, polyphenol content of foods has been shown to vary dependent on processing [50]. These discrepancies between supplement types may also exist between supplements of the same type, obtained from different places. Participants in the current study obtained their supplements from a range of sources. The quality and polyphenol content of supplements could thus differ, and therefore the dose required for effective supplementation and desired outcomes of use would also differ.

There is some evidence that whole fruit supplementation of both TC and sweet cherry may also provide beneficial effects to plasma biomarkers of inflammation in health subjects. Indeed, supplementation of 2 servings (280 g) of Bing sweet cherries (BSC) has been shown to lower plasma urate levels 5 h following consumption [51]. In addition, supplementation of 280 g BSC for 28 days has also been shown to reduce plasma biomarkers of inflammation associated with the vascular health including endothelin-1, epidermal growth factor and interleukin-8 [52]. However, it is important to note that the majority of studies investigating cherry supplementation use concentrate or juice supplements. No studies investigating sporting performance or recovery from exercise have employed a whole fruit supplementation strategy [53].

Whole fruit consumption, whilst able to provide the same effects as supplementation with concentrate or powder, is much more difficult to accurately dose, due to fruit to fruit variability; the large variability in phytochemical content between harvests related to growing conditions [54], and the relative difficulty in assessing polyphenol content compared to batches of a concentrated supplement. Furthermore, 63.3% of respondents indicated that ease of consumption or convenience of use was a major factor in their decision regarding supplement type used. This is a potential drawback to whole fruit consumption when compared to a concentrate supplement.

The wide variety in supplementation protocols within the literature illustrates the need for more studies investigating the optimal dose and supplementation protocol, to provide an evidence-based methodology that can be followed by both practitioners and athletes. Furthermore, future research should also conduct direct analysis of the polyphenol content of supplements. In the current literature, only a few studies [3,4,9,38,49] present data on the polyphenol content of the supplement used, meaning they are unable to ascertain the polyphenol dose provided. This is important, as previous research suggests that the effects observed following supplementation are dependent on polyphenol dose, whereby a dose of at least 1200 mg·d^−1^ is required to see ergogenic effects to muscle recovery [2,3,4,5,6], with studies supplementing at levels below this (480–733 mg·d^−1^) showing no effects [40,49]. However, it also appears that for performance enhancements the dose required is not as high, with a lower dose of ~465 mg·d^−1^ being shown to be effective [9]. Future studies should make the dose used clearer and companies manufacturing supplements should publish the polyphenol content of their products on packaging.

It is important to note that habitual dietary consumption of polyphenols may also be a factor in both the dose required for enhancements to be observed, and the effectiveness of supplementation. Individuals with a high habitual polyphenol intake may not require as high a dose, or may not experience beneficial effects of supplementation if their habitual intake is at a level already likely to provide beneficial effects. The relationship between habitual diet and TC supplementation is currently unclear within the literature. The majority of studies investigating the effects of TC supplementation on exercise performance or recovery either do not assess dietary polyphenol intake, or restrict dietary polyphenol intake during the study, which has obvious implications for external validity and ‘real world’ application of supplementation. Future research in this area should allow participants to maintain their habitual diet, but should attempt to assess the polyphenol content within participants’ diets to allow for insights into how this may influence the effectiveness of supplementation.

Improved recovery, sleep, and immunity and general health were the goals most frequently indicated by respondents using TC supplements, with the primary objective of supplementation clearly being improved recovery; with 71.4% of respondents using TC supplements indicating improved recovery as a goal of supplementation (Figure 2A). The current evidence base, whilst not unequivocal, is broadly supportive of TC supplementation enhancing recovery. It has been shown that TC supplementation can significantly reduce soreness, inflammatory markers and loss of strength from muscle damaging exercise [1,4,5,6,40,49], as well as enhance strength recovery [2,3,5]. The results of the current study suggest that these findings have broadly been well disseminated. However, to date only three studies have shown improvements in sleep following TC supplementation [10,11,12], only one of which showed these improvements in the current population of healthy and active adults [10]. Indeed, in the current study, only 14.3% of respondents who had previously used TC supplements indicated that they had experienced improvements in sleep. With regard to TC use for general health and immunity, there is some evidence that reductions in oxidative stress and inflammation following TC supplementation may provide some benefits for joint health [55,56], blood pressure [57], immune function [58], and neuroprotection [59,60]. However, evidence in this area is equivocal, and similarly to sleep, the majority of data in this area have not been generated from a healthy and active population. The discrepancy between use of TC supplements for sleep promotion and the current evidence for the efficacy of sleep as an application was also identified in a study of TC use by sports science practitioners [35]; which further reinforces the need for research into the effects of TC supplementation on sleep in young healthy populations.

Interestingly, the first use of TC supplements was evenly split with 50% of athletes who had used or were using TC supplements having first used TC supplements at an elite level, and 50% at a sub-elite level. Furthermore, there was no significant difference between elite and sub-elite respondents in perceived knowledge of the supplement, nor in their practical application of this knowledge with regard to dose or pre- and post-load duration. Finally, there was no significant difference between elite and sub-elite participants in the evidential support for their beliefs in the effects of TC supplementation or in the professionality of the source of their information. The lack of coalescence around any supplementation strategy by respondents is likely a result of the lack of a standardised or optimal strategy in the existing literature. Furthermore, whilst athletes at higher competitive levels may have greater access to sports science practitioners, previous research on supplementation habits of professional athletes has shown that access to information does not necessarily align with their knowledge of supplements [34]. Indeed, if, as suggested by the results of the current study, athletes at elite and sub-elite levels are getting their information from similar sources, it is unsurprising that there is little difference in the evidential support for their beliefs, or in their applications of supplementation strategies.

It is important to note too, that some research suggests that only a small percentage of the elite athlete population may actually have the opportunity to consult with a sports nutrition professional [61], and that even when consultation is available to athletes, it is not always utilised, with athletes eschewing professional consultation in favour of other sources [61,62,63]. This could perhaps explain this observed similarity in sources of information between athletes at different competitive levels, despite potential access to differing sources of information.

Supplements have been shown previously to be used extensively by the athlete population, with reasons for supplementation ranging from aiding recovery, improving general health, improvement performance, treating illness, and supplementing a perceived unbalanced diet [64,65,66,67]. One of the most commonly used have been shown to be vitamin and antioxidant supplements, minerals, protein and creatine, and other ergogenic supplements such as caffeine and ginseng [65]. TC supplementation is therefore unsurprisingly something athletes may consider using, as it falls within both the antioxidant category and there is evidence to suggest supplementation may improve recovery, performance and have general beneficial health effects. Whilst many supplements used by athletes are supported by evidence, as in the current study, athlete use has been shown previously to be inappropriate with regard to dose, timing of dose and usage of the supplement [65]. This is similar to the findings of the current study, where supplementation protocol, and athlete knowledge of literature-supported dosing strategies was shown to be an issue.

The lack of side effects experienced by respondents aligns with the literature, where there have been no reports of any side effects following TC supplementation. Furthermore, 68.8% of participants who had used or currently use TC supplements responded that they enjoy the taste, with 12.5% indicated they had no strong feelings on the taste, and only 18.8% that they do not enjoy the taste. To our knowledge, this is the first study to survey athletes on the impact of TC palatability on supplementation. These data suggest that TC supplements are a viable option for general use as a supplement.

## 5. Conclusions

TC supplementation is not widely used by athletes. The primary goals of respondents in using TC supplements were to improve recovery, sleep quality and duration, and general health and immune function. Whilst the primary goal of recovery aligns well with evidence from the literature, the secondary goals are less well supported, suggesting that whilst dissemination of the research investigating recovery may be good, there is a general lack of clarity in terms of what other benefits can be expected following supplementation, suggesting both more research is required in these areas; namely performance, sleep and immune function, and better dissemination of the subsequent findings. Choosing supplementation dose and duration were shown to be a challenge for athletes, with use of many different supplementation protocols. Athlete awareness of a literature-supported dosing strategy was poor and did not appear to be related to competitive level; however, this confusion is perhaps unsurprising, given the lack of clarity on supplementation strategy within the published literature.

Future research should focus on investigating outcomes which have associated anecdotal evidence from the athlete population (i.e., sleep duration and quality) that do not have a strong experimental evidence base; as well as on developing optimal supplementation protocols with regard to pre- and post-event TC load, TC dose timing and polyphenol/anthocyanin doses. Where possible, manufacturers should also publish the batch-specific polyphenol content of their supplement to allow for more informed dosing during supplement use. This would be especially useful in allowing athletes to modify their supplementation protocol such that it is appropriate for the desired outcome (e.g., recovery or performance), where the required polyphenol intake for ergogenic effects may differ. Scientific journals were the primary source of information for athletes, which suggests that evidence-based supplementation protocols should be more clearly outlined in the literature. Furthermore, improving the prominence of dosing considerations in the literature may help in the dissemination of information through the next three most common sources of information: web search, social media and word of mouth, as these sources are unlikely to highlight nuances outside of the ‘headline’ findings.

## Figures and Tables

**Table 1:** Participant training and competition demographics.

Type of Training	Aerobic Conditioning	Resistance Training	Speed Training	Skills Training	Mobility Exercises	-	-	-
	96.3%	90.0%	66.3%	51.3%	1.3%	-	-	-
Current Competitive Level	International	National	Professional	Semi-Professional	State/Regional	County	Club	Recreational
	5.0%	33.8%	2.5%	2.5%	6.3%	1.3%	27.5%	20.0%
Time at Current Competitive Level	<1 year	1–2 years	2–5 years	5–10 years	≥10 years	-	-	-
	12.5%	21.3%	28.8%	21.3%	15.0%	-	-	-
Highest Competitive Level	International	National	Professional	Semi-Professional	State/Regional	County	Club	Recreational
	20.0%	30.0%	2.5%	2.5%	12.5%	2.5%	16.3%	12.5%
Competition Frequency	Weekly or More	Monthly or More	6–11 Times per Year	2–5 Times Per Year	Do Not Compete	-	-	-
	27.5%	28.8%	13.8%	23.8%	6.3%	-	-	-

**Table 2:** Side effects and their corresponding severity experienced by participants (*n* = 18).

Side Effect	Severity of Side Effect				
	No Effect	Unsure	Small Effect	Noticeable Effect	Severe Effect
Gastrointestinal distress/Diarrhoea	77.8%	5.6%	11.1%	5.6%	0.0%
Weight Loss	83.3%	11.1%	0.0%	5.6%	0.0%
Increased Appetite	83.3%	11.1%	0.0%	5.6%	0.0%
Decreased Appetite	94.4%	5.6%	0.0%	0.0%	0.0%
Increased thirst/fluid consumption	77.8%	11.1%	5.6%	0.0%	5.6%
Bruising under the skin	83.3%	11.1%	5.6%	0.0%	0.0%
Dizziness	94.4%	5.6%	0.0%	2.0%	0.0%
Joint Pain	88.9%	11.1%	0.0%	0.0%	0.0%

## Data Availability

The raw data for this study are available online with the Supplementary Materials S2.

## Supplementary Materials

The following are available online at https://www.mdpi.com/article/10.3390/sports9040049/s1, Questionnaire (Supplementary Materials S1): ‘Attitudes towards and use of tart cherry supplementation by athletes’.
