# Supplementary material for: Use, Practices and Attitudes of Elite and Sub-Elite Athletes towards Tart Cherry Supplementation
Source: Sports (Basel). 2021 Mar 31;9(4):49. doi: 10.3390/sports9040049 (PMC8066185; doi:10.3390/sports9040049)
Supplement: Supplementary file 1 [file sports-09-00049-s001.zip › sports-1131038-supplemantary materials/sports-1131038-supplementary materials 1.pdf]

Welcome, Information and Consent

Thank you for following this link to participate in the research study titled Attitudes towards and use of tart cherry supplementation by sports nutrition; strength & conditioning practitioners, and athletes. This study is being done by Vlad Razvan Sabou at the University of Exeter and Jimmy Wangdi at the University of Exeter and University of Queensland.

This study is investigating the current use, practices and attitudes of athletes from a variety of competitive levels on tart cherry supplementation. Tart Cherry supplementation has received a high degree of scientific interest over the last decade, but there is little published data about current approaches it's supplementation in applied sport science practice.

If you are interested in taking part, please read the participant information sheet and keep a copy before starting the questionnaire. If you have any questions, please email me at j.wangdi@uq.net.au. Your participation is entirely voluntary, and you can withdraw at any time.

Please take your time to read through the attached information sheet. If you are still happy to participate in the study after reading through the information, please fill out the consent form.

Participant Information Sheet

CONSENT FORM

Title of Project: Attitudes towards and use of tart cherry supplementation by sports nutrition; strength & conditioning practitioners, and athletes.

Name of Researchers: Mr Vlad Razvan Sabou, Dr Mary O'Leary and Professor Joanna Bowtell, Mr Jimmy Wangdi, Dr Vincent Kelly

Informed Consent Form for Participants

I confirm that I have read the information sheet dated 10/04/2020 (version no1) for the above project. I have had the opportunity to consider the information, ask questions and have had these answered satisfactorily.

- Yes
- No

I understand that my participation is voluntary and that I am free to withdraw at any time without giving any reason and without my legal rights being affected.

- Yes
- No

I understand that relevant sections of the data collected during the study may be looked at by members of the research team and individuals from the University of Exeter and University of Queensland, where it is relevant to my taking part in this research. I give permission for these individuals to have access to my records.

- Yes
- No

I understand that taking part involves identifiable questionnaire responses.

- Yes
- No

I understand that my data will be used for the purposes of:

- Being included in a digital database for up to 5 years
- Anonymised data may be published in academic or lay science publications
- Anonymised data may be used in teaching or training materials for use in University or public engagement activities.
- I agree that my contact details can be kept securely and used by researchers from the study team to contact me about future research projects

I agree to take part in the above study.

- Yes
- No

Click to write the question text

× SIGN HERE

clear

Thank You for providing your consent, you can now proceed to the questionnaire.

Section 1: Demographic-related questions/statements:

Please indicate your sex

Male	Female	Would Rather Not Say
○	○	○

Please indicate your age (years)

Please indicate your height (cm)

Please indicate your body mass to the nearest kilogram.

What main sport do you compete in? Please write your answer in the box below:

Do you compete in any other sports? Please write your answer in the box below:

What type of training is involved in the training for this sport? Select all that apply

- [ ] Resistance Training e.g. weights, strength work
- [ ] Aerobic Conditioning e.g. running, cycling, boxing ergometer work
- [ ] Speed Training
- [ ] Skills Training
- [ ] Other (please elaborate):

Please indicate the country in which you predominantly complete/train:

What is your current competitive level within your sport?

- International
- National
- State/Regional
- County
- Club
- Recreational
- Professional
- Semi-Professional

What is the highest competitive level at which you have competed?

- International
- National
- State/Regional
- County
- Club
- Recreational

- Professional
- Semi-Professional

How long have you competed at your current competitive level?

- <1 Year
- 1-2 years
- 2-5 Years
- 5-10 years
- ≥10 Years

How many hours (to the nearest hour) per week do you spend training? (Please note, as stated in the participant information sheet, you must complete at least 4 hours per week to participate in this study)

	0	5	10	15	20	25	30	35	40	45	50	55	60
Hours Per Week													

How often do you compete in your main sport?

Section 2. Use of tart cherry supplements as part of applied practice:

How would you rate your knowledge (awareness, potential benefits, when to take it, how much to take, etc.) of tart cherry supplements?

- Excellent
- Good
- Average

- ○ Limited
- ○ No Knowledge

What sources have you used to find/received information on tart cherry supplements? Choose all that apply.

- ☐ Social Media (Twitter/Instagram/YouTube/etc.)
- ☐ Scientific Journals
- ☐ News/Media Sources (Newspaper/TV/Magazines/etc.)
- ☐ Web Search
- ☐ Podcasts/Blog Subscription
- ☐ Word of Mouth (e.g. team mates)
- ☐ Company Marketing (e.g. adverts)
- ☐ Doctor/Physician/Healthcare Professional
- ☐ Team Staff Member (Coach/Sports Nutritionist/Dietitian/Sports Scientist/etc.)
- ☐ Health Food Store
- ☐ ________ Other (please elaborate)

What effects do you think tart cherry supplementation can have on aspects of athletic performance? Choose all that apply.

- ☐ Improved post-exercise recovery
- ☐ Improved sleep quality and duration
- ☐ Improved endurance exercise performance
- ☐ Improved repeated sprint performance
- ☐ Improved strength performance
- ☐ Enhanced training adaptations
- ☐ Enhanced immunity and general health
- ☐ Enhanced muscle mass development
- ☐ Enhanced injury rehabilitation
- ☐ No effect

- ☐ [ ] Others (please elaborate)

Have you used/do you plan on using tart cherry supplements?

- ◯ I currently use tart cherry supplements
- ◯ I have previously used tart cherry supplements, but no longer do so
- ◯ I have not used tart cherry supplements before, but I plan on doing so in the future
- ◯ I have never used tart cherry supplements and do not plan to do so

What are/were your reasons for not using tart cherry supplements? (select all that apply)

- ☐ Personal/religious beliefs
- ☐ No knowledge of supplements
- ☐ The cost is too high
- ☐ I am healthy enough
- ☐ Tart cherry has no effect
- ☐ I am afraid of the side effects of tart cherry
- ☐ I am afraid of doping issues
- ☐ [ ] Other (please elaborate)

When was the last time you used a tart cherry supplement?

- ◯ Past 7 days
- ◯ Past 30 days
- ◯ Past 1-6 months
- ◯ Past 7-12 months
- ◯ >1 year ago

At what level were you competing when you first used tart cherry supplementation, in either training or competition?

- ○ International
- ○ National
- ○ State/Regional
- ○ County
- ○ Club
- ○ Recreational
- ○ Professional
- ○ Semi-Professional

What is/was your goal in using tart cherry supplements? Please indicate all the applicable answers.

- ☐ Improved post-exercise recovery
- ☐ Improved sleep quality and duration
- ☐ Improved endurance exercise performance
- ☐ Improved repeated sleep performance
- ☐ Improved strength performance
- ☐ Enhanced training adaptations
- ☐ Enhanced immunity and general health
- ☐ Enhanced muscle mass development
- ☐ Enhanced injury rehabilitation
- ☐ ________ Other (please elaborate)

What type of tart cherry supplement do you/would you use? Please indicate all the applicable answers

- ☐ Tart cherry concentrate juice
- ☐ tart cherry juice

- [ ] Tart cherry powder
- [ ] tart cherry gel
- [ ] ______ Other (please elaborate)

Where do you/would you obtain your tart cherry supplement? Select all that apply.

- [ ] Supplement store
- [ ] Health food store
- [ ] Online store
- [ ] Provided by team/club staff
- [ ] ______ Other (please elaborate)

How do you/would you obtain your tart cherry supplement? Select all that apply.

- [ ] I purchase it
- [ ] I am provided it by a team sponsor
- [ ] I was provided it by an individual sponsor
- [ ] ______ Other (please elaborate)

How do/did/would you use tart cherry supplement?

- ○ Acute supplementation (one off dose 1-2hrs pre-competition)
- ○ Chronic supplementation (longer term use over a period of days or weeks)
- ○ Both acute and chronic supplementation

What is/was/would be the usual duration of your supplementation protocol/use? Please indicate all appropriate answers

- ○ 2-3 days
- ○ 3-5 days
- ○ 5-8 days
- ○ Continuous Use

What is the main source you used for informing your supplementation protocol (dose/length of time/etc.)?

- ○ Relevant literature
- ○ Manufacturers recommendations
- ○ Discussion with academics involved in tart cherry/polyphenol research
- ○ Sports dietitian / Sports Nutrition practitioners
- ○ Strength and conditioning practitioners
- ○ Web Search
- ○ __________ Other (please elaborate)

What dose of tart cherry supplement to do/did/would you use for chronic supplementation? Write in the mL/number of tablets per day and the name of the brand:

____

Are you aware of the polyphenol amount contained within this dose? If so please write in here:

____

What dose of tart cherry supplement to do/did/would you use for acute supplementation? Write in the mL/number of tablets per day and the name of the brand:

____

Are you aware of the polyphenol amount contained within this dose? If so please write in here:

During which periods do/did/would you use tart cherry supplements? Please indicate all appropriate answers

- ☐ Before and after competition
- ☐ During periods with multiple condensed athletic events
- ☐ During pre-season or demanding training blocks
- ☐ Prior to specific training sessions
- ☐ Following specific training sessions
- ☐ Daily basis

What is/was/would be the usual duration of your pre-exercise (training or competition) supplementation protocol if using tart cherry supplements for enhancing recovery?

- ○ 1 day prior to exercise
- ○ 2 days prior to exercise
- ○ 3 days prior to exercise
- ○ 4-5 days prior to exercise
- ○ 6-8 days prior to exercise
- ○ Not Applicable
- ○ Other (please elaborate)

What is/was/would be the usual duration of your post-exercise (training or competition) supplementation protocol if using tart cherry supplements for enhancing recovery?

- ○ 1 day following exercise
- ○ 2 days following exercise

- 3 days following exercise
- 4-5 days following exercise
- Not Applicable
- Other (please elaborate)

Have you experienced any side effects of supplementation? Please indicate all appropriate answers:

	Severity				
	No Effect	Unsure	Small Effect	Noticeable Effect	Severe Effect
Gastrointestinal distress/Diarrhoea	○	○	○	○	○
Weight Loss	○	○	○	○	○
Increased Appetite	○	○	○	○	○
Decreased Appetite	○	○	○	○	○
Increased thirst/fluid consumption	○	○	○	○	○
Bruising under the skin	○	○	○	○	○
Dizziness	○	○	○	○	○
Joint Pain	○	○	○	○	○
Other (please elaborate)	○	○	○	○	○

If you selected other in the previous question, please elaborate here, including the severity:

Section 3: Attitudes towards tart cherry supplements

Do you believe that the current literature supports any of the following beneficial effects for tart cherry supplementation? Please indicate all appropriate answers.

- ☐ Improved post-exercise recovery
- ☐ Improved sleep quality and duration
- ☐ Acute supplementation improves exercise performance
- ☐ Enhanced immunity
- ☐ Enhanced injury rehabilitation
- ☐ None of the above
- ☐ I am unsure

For which type of sports do you believe the literature supports the ergogenic effects (benefits to performance) of acute tart cherry supplementation? Please indicate all the applicable answers

- ☐ Strength-based sports
- ☐ Endurance-based sports
- ☐ Power-based sports
- ☐ Intermittent-running based sports (e.g. team sports)
- ☐ None of the above
- ☐ ________ Other (please elaborate)
- ☐ I am unsure

Please indicate in a short sentence if you have noticed any improvements in your performance following tart cherry supplementation

Please indicate in a short sentence if you have noticed any improvements in your recovery

following tart cherry supplementation

Please indicate in a short sentence if you have noticed any improvements in your sleep following tart cherry supplementation

How do you believe tart cherry supplementation may affect improvements from training?

- Likely to have a positive effect
- Likely to have a negative effect
- Likely to have no effect
- Do not have an opinion of this

If tart cherry supplementation was shown to impair improvements from training, would you still use it and if so how?

- I would not use it
- I would still use it consistently
- I would use one off acute doses for performance
- I would use chronic doses during specific periods of training/off season
- I would use chronic doses during specific periods of competition
- Other

What were your biggest considerations when deciding on how to develop your tart cherry supplementation protocol?

- ☐ Current literature shows mixed findings regarding the efficacy of the supplement
- ☐ Lack of clarity regarding the existence of an optimal supplementation protocol (duration & dose)
- ☐ Lack of research illustrating the effects on training adaptations
- ☐ Difficulties in choosing between available products on the sports supplements market
- ☐ Supplement pricing
- ☐ Prioritising other supplements (Please indicate which supplements):
- ☐ [ ] Others (please elaborate)

What is your opinion of the taste of cherry juice?

- ○ I enjoy the taste
- ○ I do not enjoy the taste
- ○ I have no strong feelings on the taste

Did your experience of the taste influence your decision to use the supplement?

- ○ Yes
- ○ No

What type of tart cherry product would you like to see developed in the future? Please briefly describe why.

- ○ Beverage
- ○ Concentrate
- ○ Gel
- ○ Bar

○ [ ] Other

What are your reasons behind your answer to the last question?

[ ]

Powered by Qualtrics
